# Could retrograde reperfusion combined with washout technique broaden the applicability of marginal grafts in liver transplantation? Intra-operative and short-term outcomes of a prospective cohort

**DOI:** 10.1590/0100-6991e-20233489-en

**Published:** 2023-06-22

**Authors:** OLIVAL CIRILO, LUIZ EDUARDO RAFAEL MOUTINHO, PAULO SÉRGIO VIEIRA DE MELO, LUDMILA RODRIGUES COSTA, PRISCYLLA JENNIE MONTEIRO RABÊLO, AMERICO GUSMÃO AMORIM, CLÁUDIO MOURA LACERDA MELO

**Affiliations:** 1 - Hospital Universitário Oswaldo Cruz, Unidade de Transplante de Fígado - Recife - PE - Brasil; 2 - Universidade de Pernambuco, Faculdade de Ciência Médicas - Recife - PE - Brasil

**Keywords:** Liver Transplantation, Reperfusion Injury, Bile Ducts, Donor Selection, Transplante de Fígado, Reperfusão, Ductos Biliares, Seleção do Doador

## Abstract

**Introduction:**

many revascularization techniques were designed to reduce the imbalance of ischemia-reperfusion injury. This study’s objective is to evaluate retrograde reperfusion (RR) compared to sequential anterograde reperfusion (AR), with and without the washout technique (WO).

**Method:**

this prospective cohort study collected data from 94 deceased donor orthotopic liver transplants and divided it into three groups: RR with WO (RR+WO), AP with WO (AR+WO), and AP without WO (AR). This study did not assign the reperfusion technique to the participants. The primary outcome considered the early graft dysfunction, and secondary outcomes included post-reperfusion syndrome (PRS), post-reperfusion lactate, surgery fluid balance, and vasoactive drug dose during the surgery.

**Results:**

*87* patients were submitted to the final analysis-29 in the RR+WO group, 27 in the AR+WO group, and 31 in the AR group. Marginal grafts prevalence was not significantly different between the groups (34% vs. 22% vs. 23%; p=0.49) and early graft dysfunction occurred at the same rate (24% vs. 26% vs. 19%; p=0.72). RR+WO reduced serum post-reperfusion lactate (p=0.034) and the incidence of significant PRS (17% vs. 33% vs. 55%; p=0.051), but norepinephrine dosing >0.5mcg/kg/min were not different during the surgery (20,7% vs. 29,6% vs. 35,5%, p=0.45*).*

**Conclusions:**

primary outcome was not significantly different between the groups; however, intraoperative hemodynamic management was safer using the RR+WO technique. We theorized that the RR+WO technique could reduce the incidence of PRS and benefit marginal graft survival following diseased donor orthotopic liver transplantation.

## Abstract (pt)

**Introdução:**

várias técnicas de reperfusão foram desenvolvidas a fim de reduzir o dano da lesão induzida por isquemia-reperfusão. Este estudo objetivou avaliar a reperfusão retrograda (RR) comparado com a reperfusão anterógrada (AR), com e sem a realização da técnica de lavagem do enxerto (WO).

**Métodos:**

coorte prospectiva com 94 transplantes ortotópicos de fígado de doador falecido divididos em três grupos: RR com WO (RR+WO), reperfusão anterógrada com WO (AR+WO), e AR sem WO (AR). Este estudo não designou a técnica de reperfusão entre os participantes. O desfecho primário considerou a disfunção precoce do enxerto, e os desfechos secundários incluíram a síndrome pós-reperfusão (SPR), lactato pós-reperfusão, balanço hídrico operatório, e uso de drogas vasoativas durante o ato peratório.

**Resultados:**

87 pacientes foram submetidos para consolidação dos dados-29 no RR+WO, 27 no AR+WO, e 31 no AR. A prevalência de enxertos maginais não diferiu entre os grupos (34% vs 22% vs 23%; p=0,49). Disfunção precoce do enxerto ocorreu em uma proporção similar (24% vs 26% vs 19%; p=0,72). RR+WO reduziu o lactato sérico pós-reperfusão (p=0,034) e a incidência de SPR severa (17% vs 33% vs 55%; p=0,051), entretanto a infusão de noradrenalina >0,5mcg/kg/min não foi diferente durante a cirurgia (20,7% vs 29,6% vs 35,5%, p=0,45).

**Conclusões:**

o desfecho primário não diferiu significativamente entre os grupos; entretanto, o manejo hemodinâmico intra-operatório foi mais seguro no grupo RR+WO. Nós teorizamos que a técnica RR+WO pode reduzir a SPR e beneficiar enxertos marginais no transplante de fígado.

## INTRODUCTION

Liver transplantation (LT) is the most effective treatment for end liver disease. The outcome of this procedure has significantly improved over the past decade; however, the reperfusion phase remains a weak spot of this procedure^1^. Ischemia-reperfusion injury releases vasoactive proinflammatory mediators and shifts the intravascular volume to the third space^2^. This phenomenon results in dramatic hemodynamic events, such as hypotension, bradycardia, or dysrhythmias called Post-Reperfusion Syndrome (PRS)^3^. 

Many revascularization techniques in LT have been described to alleviate the PRS and favor graft survival^4^. The current literature reports different approaches as: 1) initial portal reperfusion, 2) initial hepatic artery reperfusion, 3) simultaneous portal and hepatic artery reperfusion, and 4) retrograde inferior cava vein (ICV) reperfusion. 

The portal vein (PV) unclamping with anterograde reperfusion (AR) was the first technique developed by Starzl et al. using conventional surgery with venovenous bypass. It has been widely used in transplant centers. It is based on physiological precepts of liver vascularization^5^. The significant hepatic inflow is through the portal vein, and early portal vein unclamping reduces intestinal edema. For these reasons, it is reasonable to assume that AR with portal vein unclamping downgrades warm ischemia duration and PRS.

However, the first idea of anterograde portal reperfusion may oppose another complex pathomechanism of the PRS that has yet to be understood. Abrupt high blood flow can, paradoxically, aggravate organ perfusion. It is called ‘paradoxical reperfusion’ and yields massive reactive oxygen species that cause endothelial inflammation and capillary occlusion^6^. 

The retrograde revascularization (RR) technique was proposed in 2003; it constitutes a reperfusion technique that provides low and gradual backflow of blood to the graft^7^. The low-oxygenated blood of IVC attenuates the post-reperfusion load of oxygen free radicals and reduces circulatory and electrolyte imbalances^8^. 

Few studies have proposed to understand RR techniques and their effects on intraoperative hemodynamics^6^ ^,^ ^7^ ^,^ ^9^ ^,^ ^10^. Most studies compared sequentially versus simultâneos AR^11^ ^,^ ^12^. RR technique has been investigated since, but it still lacks more original data and uniformization. Therefore, we designed this study to evaluate RR compared to sequential AR, with and without washout technique (WO), in terms of intraoperative hemodynamic stability, electrolyte imbalances, and early graft function in deceased LT.

## PATIENTS AND METHODS

This prospective cohort study followed deceased donor LT from December 2018 to January 2020 at the University Hospital Oswaldo Cruz Pernambuco, Brazil. We divided the patients into three groups: retrograde reperfusion with washout technique (RR+WO), anterograde reperfusion with washout technique (AR+WO), and anterograde reperfusion without washout technique (AR). 

This study method did not assign the reperfusion technique. Instead, it was an intraoperative decision of the assistant surgeon based on his technical experience and clinical judgment. The team of this investigation recorded, prospectively, pre-defined data until the seventh-day post-transplant. The primary outcome was early graft dysfunction, and secondary outcomes included post-reperfusion syndrome, post-reperfusion lactate, surgery fluid balance, and vasoactive drug dose during the surgery. 

Adult patients with end-stage liver disease were included. We excluded patients with: fulminant liver failure, combined transplantation, or split graft. No other criteria were applied to exclude any participant. Comorbidities (systemic arterial hypertension, diabetes mellitus), PV thrombosis, and MELD-Na were considered risk factors for the recipient.

The conventional hepatectomy technique was performed and both portal flow and vena cava flow were interrupted, whereas the piggyback approach only occluded portal flow. The surgeon’s preference defined it. No bypass was used in the conventional technique. Modified retrograde reperfusion was performed by IVC supra and infra-hepatic anastomoses and, as soon as it was completed, backward unclamping. During the anastomosis of the infra-hepatic IVC, the wash-out technique was carried out by the infusion of 1000ml of frozen saline 0,9% solution through the PV. Following retrograde reperfusion, 100ml of blood was released through the PV into the cavity. Next, PV anastomosis was performed and then unclamped. Then, hepatic artery anastomosis was done. Anterograde reperfusion was carried out by the following steps: conventional (or piggyback) hepatectomy; IVC supra (and infra-hepatic) anastomoses; PV anastomosis; portal and IVC supra (and infra-hepatic) unclamp; hepatic artery anastomosis and hepatic artery unclamp. Wash-out in anterograde reperfusion was performed in the same way as in retrograde reperfusion and blood release was done throughout the infra-hepatic IVC after PV unclamp^7^.

Warm ischemia was defined as the interval from removal from cold storage to the establishment of reperfusion of the liver graft either through hepatic veins or portal vein. While portal ischemia was defined as the interval from removal from cold storage to establishing portal vein unclamp. 

We used the Histidine-Tryptophan-Ketoglutarate (HTK) solution at 4ºC to preserve the graft. 

The deceased donor was defined as expanded criteria liver donor - marginal grafts - with at least one of: age over 60 years, length of ICU stay over four days, bilirubin over 2,0mg/dl, AST over 170U/I, ALT over 140U/I, bloodstream sodium over 155mEq/l and macrosteatosis over 30%, need for vasopressor drugs (use of a dose >10µg/kg/min of dopamine or >0,5µg/kg/min of noradrenaline).

The hemodynamic outcome was evaluated in three domains: ischemia-reperfusion syndrome, microvascular hemodynamic, and macrovascular hemodynamic. Mild PRS was defined by a decrease of mean arterial pressure or heart rate fewer than 30% of baseline value that lasts for less than 5 minutes and that responsive to an intravenous bolus dose of Calcium Chloride (1g) or epinephrine (≤100mcg). Significant PRS was defined by a drop of mean arterial pressure or increase of heart rate greater than 30% of baseline, asystole, or hemodynamically significant arrhythmias; or the need to start the infusion of vasopressors that persist throughout the postoperative period. Absent PRS was assigned when none of these alterations were observed^2^. Two subsequent arterial gasometry [pH, pCO_2_ (mmHg), lactate (mmol/L) and potassium (mmol/L)] were used to measure microvascular dysfunction the first, 30 minutes before reperfusion, and the second, 30 minutes after reperfusion. Finally, the macrohemodynamic evaluated the need to maintain vasoactive amines at the end of the surgery and the fluid intake during the surgery. 

Early graft dysfunction (EGD) was defined within at least two of the following criteria during the first seven days: ALT over 2000U/l, bilirubin over 10mg/dl, or INR over 2,0. Primary non-function (PNF) was defined within EGD associated with retransplantation or death after hepatic thrombosis artery was excluded. 

Statistical data analysis was done in SPSS^®^ Statistics 13.0 (IBM^®^ Inc.). The data values were presented as absolute frequency plus relative frequency and median plus interquartile range. All inferential tests performed were two-tailed. All conclusions were taken at the 5% significance level.

This study was approved by the Human Research Ethics Committee of the institution where it was conducted (Ethical approval number: 21092619.1.0000.5201), following the principles of the Brazilian National Health Council.

## RESULTS

During the studied period, 87 patients were submitted to the final analysis 29 in the RR+WO group, 27 in the AR+WO group, and 31 in the AR group. Seven patients were excluded: four patients were excluded because they did not undergo the washout technique combined with RR and three patients were excluded from lack of postoperative data.

We summarized receptor and donor baseline characteristics in Table 1. In the study population, the male gender in the fifth decade of life suffering from alcohol-related cirrhosis prevailed. Receptor risk factors were similar, and marginal grafts’ prevalence did not differ between RR+WO, RA+WO and RA (34% vs. 22% vs. 23%; p=0.49). Conventional LT without venovenous bypass was performed in 90% of the patients submitted to RR+WO. Surgery duration, cold ischemia, portal ischemia, and warm ischemia were all significantly lower in the RR+WO group. Intraoperative outcomes showed no difference in bile production, MAP at the end of the surgery, temperature, crystalloids, and blood-derivatives reposition. High dosing of vasoactive agents during the surgery did not statistically differ between the groups (20.7% vs. 29.6% vs 35.5%; p=0,45). PNF incidence did not show a statistical difference between the groups (17% vs. 11% vs. 10%; p=0.66). EGD was similar between the groups (24% vs. 26% vs. 19%; p=0.72). 

**Table 1:** Characteristics of the Study Population and Early Outcomes.

Variables*	RR + WO		AR + WO			AR	p-value**
n	29	27	31			31	
Gender							
Male	21	72%	19	70%	21	68%	
Female	8	28%	8	30%	10	32%	0.956
Age. y	56	(43-60)	58	(40.5-62.5)	53	(33.5-63)	0.645***
BMI*	25.5	(23.6-27.3)	24.7	(23.7-26.8)	24	(21.3-27.1)	0.382***
Cause of LT							
Alcohol	12	41.4%	6	22.2%	8	25.8%	
HCV	2	6.9%	5	18.5%	4	12.9%	
Variables*	RR + WO		AR + WO			AR	p-value**
NASH	1	3.4%	1	3.7%	4	12.9%	
Cryptogenic cirrhosis	2	6.9%	5	18.5%	4	12.9%	
Autoimmune hepatitis	3	10.3%	2	7.4%	2	6.5%	
Schitossomosis	3	10.3%	0	0.0%	0	0.0%	
Primary sclerosing cholangitis	2	6.9%	0	0.0%	4	12.9%	
Outros	4	13.8%	8	29.6%	5	16.1%	
Risk Factors							
DM	5	17%	5	19%	4	13%	0.874
HAS	5	17%	9	33%	6	19%	0.314
Thrombosis PV	1	3%	2	7%	5	16%	0.235
MELD-Na*	19	(17-24)	21.5	(18-25)	21	(15-26)	0.680***
Donor graft							
Standard	18	62%	20	74%	24	77%	
Marginal	10	34%	6	22%	7	23%	0.485
Surgery technique							
Conventional without VVB	26	90%	12	44%	19	61%	
Piggyback	3	10%	15	56%	12	39%	0.001
Duration* (minutes)							
Surgery	305	(280-355)	360	(325-413)	345	318-388)	0.027***
Cold ischemia	376	(295-477)	463	(377-543)	404	(354-538)	0.049***
Portal ischemia	33	(29-36)	39	(35-43)	35	(31.5-44)	0.013***
Warm ischemia	20	(18-23)	39	(35-43)	35	(31.5-44)	< 0.001***
Bile production	26	90%	26	96%	25	81%	0.198
MAP (mmHg)	70.5	(65-73)	70	(65-75)	71	(64-80)	0.775***
Norepinephrine							
> 0.5mcg/kg/min	6	20.7%	8	29.6%	11	35.5%	0.446
Temperature (ºC)	36.2	(35.7-36.9)	35.9	(35.4-36.6)	36	(35.0-36.6)	0.233***
Fluid Balance							
Packed red blood (unit)	2	(0-5)	2	(0-3)	2	(0.5-3)	0.396***
Plasma (unid)	0	(0-2)	0	(0-0.5)		0(0-0)	0.069***
Cristaloid (ml)	5,000	(4,000-7,000)	5,000	(4,250-5,950)	4,875	(4,000-5,675)	0.753***
Early graft function							
PNF	5	17%	3	11%	3	10%	0.659
EAD	7	24%	7	26%	6	19%	0.721

Patients​‘​ baseline characteristics that were submitted to liver transplant divided by reperfusion technique. Intraoperative data and early outcomes were also reported. Some confounders as marginal graft and surgery technique might disclose an unbalanced sample allocation. Surgery duration and warm ischemia were significantly lower in the RR+WO strategy. There was no significant difference in the graft function between the reperfusion strategies.​ ​RR: retrograde reperfusion​;​ WO: washout technique​;​ AR: anterograde reperfusion​;​ LT: liver transplant​;​ HCV: hepatitis C virus​;​ NASH: nonalcoholic steatohepatitis​;​ DM: diabetes mellitus​;​ PV: portal vein​;​ VVB: venovenous bypass​;​ MAP: mean arterial pressure; PNF: primary non-function; EAD: early graft dysfunction​;​ PRS: post-reperfusion syndrome​.​ *Variables presented as absolute + relative frequency OR median + (IQR)​;​ **Chi-squared test (or Fisher’s exact test)​;​ *** Kruskal-Wallis test.

**Figure 1:**
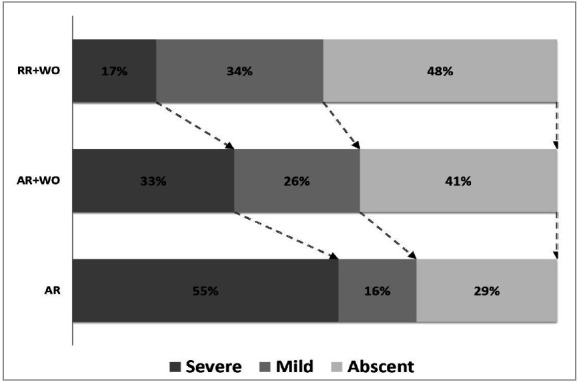
Post-reperfusion Syndrome. Association between PRS grade at the deceased donor LT and the reperfusion technique. PRS severity increases among the groups. PRS was worse in the AR and better in the RR+WO (p=0.051). Chi-squared test.​ PRS: post-reperfusion syndrome​;​ RR: retrograde reperfusion​;​ WO: washout technique;​ AR: anterograde reperfusion​;​ LT: liver transplant.

RR+WO technique minimized the prevalence of significant PRS compared to AR+WO and AR (17% vs. 33% vs. 55%; p=0.051). (Figure 1).

**Figure 2:**
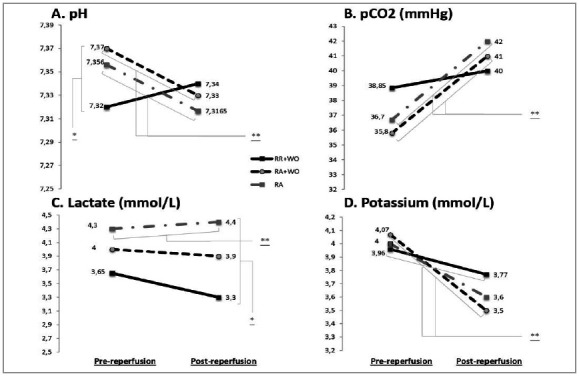
Intraoperative Stability​. Metabolic assessment of pre- and post-reperfusion liver transplantation receptor. Two subsequent arterial gasometry (30 minutes before reperfusion, and 30 minutes after reperfusion) were performed and compared between three different reperfusion strategies. A. shows an increase of post-reperfusion acidemia in the AR+WO (p=0.004) and AR (p=0.026) groups and a baseline acidosis pre-reperfusion in the RR+WO group (p=0.049). B. shows an increase in post-reperfusion hypercapnia in AR+WO (p=<0.001) and AR groups (p=<0.001). C. shows lower serum post-reperfusion lactate in the RR+WO group (p=0.034). D. shows a reduction of kalemia in the RR+WO (p=0​.​020) and the AR+WO (p=0​.​001) groups that were not observed in the AR group (p=0.085). RR: retrograde reperfusion​;​ WO: washout technique​;​ AR: anterograde reperfusion;​ Variables presented by mean. *Kruskal-Wallis tests that were significantly different. **Wilcoxon tests that were significantly different.

Intraoperative arterial gasometry before reperfusion and after reperfusion of the graft showed: 1) an increase of post-reperfusion acidemia in AR+WO (p=0.004) and AR (p=0.026) and hypercapnia in AR+WO (p≤0.001) and AR (p≤0.001); 2) lower serum post-reperfusion lactate in RR+WO (p=0.034), 3) reduction of kalemia in the RR+WO (p=0,020) and the AR+WO (p=0,001) (Figure 2). Post-reperfusion acidemia was not statistically different between the groups (p=0.288). Serum lactate pre-reperfusion did not differ between the groups (p=0.219). There was no statistical reduction of serum potassium gasometry before reperfusion and after reperfusion of the graft in the AR group (p=0.085).

**Figure 3:**
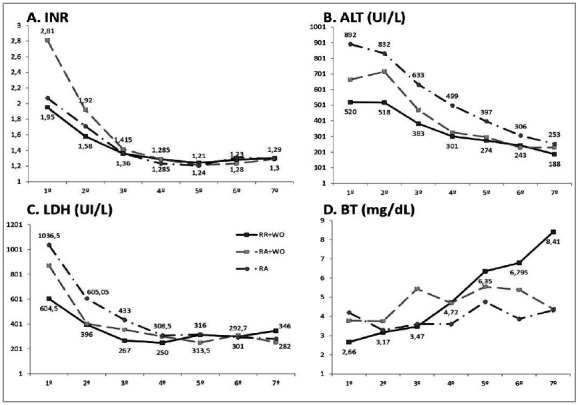
Early Graft Function. Post-transplant liver function. Post-transplant liver enzymes and INR were slightly lower in the RR+WO group, followed by the AR+WO group, and, finally, by the AR group which showed higher proportions of these variables. No significant difference was demonstrated by the statistical method. Both parameters converged on the seventh-day post-transplant between all reperfusion techniques analyzed in this study. RR: retrograde reperfusion; WO: washout technique; AR: anterograde reperfusion; INR: international normalized ratio; ALT: Alanine transaminase; LDH: lactate dehydrogenase; BT: bilirubin total; Kruskal-Wallis test did not demonstrate statistical difference (p-value >0,05).

The statistical method showed no significant difference in post-transplant liver enzymes and INR. Both parameters converged on the seventh-day post-transplant. The serum bilirubin level in the RR+WO group increased (Figure 3).

## DISCUSSION

The data of this study associate RR with lower PRS incidence. PRS is a critical moment and can affect up to 32% of the patients submitted to LT^3^. Heidenhain et al. reported 3.6% of PRS following RR and argued, as well as other authors, that RR could reduce hemodynamic instability following the graft reperfusion in the LT^9^. 

However, no definitive consensus exists that RR could produce better graft function and recipient survival outcomes. Since the first large case series of RR in 2003, this technique downgrade liver transaminases in early post-transplantation^7^ ^,^ ^10^. Only one prospective study randomized 131 LT among RR versus simultaneous portal and hepatic artery AR to test the hypothesis that RR might reduce the ischemia-reperfusion injury to the liver graft. It showed no episodes of PNF of the liver graft submitted to RR compared to 7,7%. Transaminases converged in both groups around the fifth-day post-transplant, as observed in this study^9^.

Our study did not observe differences in the early graft function between the groups. We imply this finding to the unbalanced sample submitted to different reperfusion techniques. A senior liver surgeon carried the reperfusion technique option. RR and WO techniques were used in qualitative inferior grafts and donors. It quantitatively explains marginal prevalence in the RR+WO group (34%). The unbalance of the sample limits the comparative analysis of this research; however, it highlights the role of RR+WO in sparse contexts of ideal grafts: in our sample, RR showed similar outcomes compared to AR despite the inferior graft scenario. 

However, the benefits of the hepatocytes occur at the expense of biliary ducts. Ischemia-type biliary lesions were higher in the group submitted to RR in the Heidenhain et al. study^9^. It seems to be related to the arterial predominance of the biliary epithelium perfusion devalued in the RR technique. Total bilirubin increased in the RR+WO group. We did not investigate long-term cumulative biliary complications, but we attributed the increment of total bilirubin to the detrimental effect of RR on the biliary tract.

Intraoperative management of LT is complex and exposes the patient to hemodynamic and biochemical changes. It represents a significant cause of cardiac arrest and might be evitable by the RR. In many investigations, RR was associated with a better post-reperfusion hemodynamic profile and better short-term outcomes^7^ ^,^ ^9^ ^,^ ^10^ ^,^ ^13^. Fukuwasa et al. retrospectively analyzed 313 RR LT versus 165 AR LT and showed a statistically significant reduction of cardiac arrest events by propensity score matching method (0.6% versus 4.9%, respectively)^13^.

The WO technique is another way to improve post-reperfusion hemodynamics. Kalemia was reduced in the RR+WO and the AR+WO groups, and it was not observed in the AR group. It resembles to be associated with washing out the solution in the graft before revascularization. However, the HTK solution has a relatively low potassium concentration compared to other preservative solutions employed in the transplant. The systemic spread of cytokines and eicosanoids can also be minimized by this technique^14^.

The conventional technique without venovenous bypass using AR is the standard technique performed at this transplant center. Surgery duration was still lower in the RR+WO. We do not justify it based only on the surgery technique. Other factors not pre-emptively hypothesized by this study could explain it, such as biliary reconstruction, hepatectomy technique (conventional vs. piggyback), portal vein thrombosis, obesity and post-reperfusion coagulopathy. Indeed, the applicability of RR was workable and did not jeopardize the surgical performance of this transplantation group.

This study’s primary and secondary outcomes should not vary depending on the surgical technique (conventional without venovenous bypass or Piggyback). A previous study at this center showed equivalent results between the two surgical techniques, and both have been carried out since without differing overall morbimortality^15^. 

We could perform RR with no delay to portal reperfusion. According to the definitions of this work, warm ischemia time and portal ischemia time are different. However, both are the same when performing sequential AR. The warm ischemia time is inherently reduced when RR is carried out; however, the portal ischemia time, which could have been compromised, was also reduced. 

The allocation criteria of the reperfusion technique impose important limitations on the study. This analysis observes different reperfusion techniques applied by a specific team of transplant surgeons and does not randomly assign them. Therefore, the implication of these results must be taken with due merit of the methodology. The small number of patients also implies limitations; however, the sample is appropriate for the literature in terms of liver transplantation.

## CONCLUSION

RR+WO technique combined physiopathological mechanisms that might reduce the impact of reperfusion-ischemia injury in the LT. The primary outcome was not different between RR+WO, RA+WO and RA. Secondary outcomes showed that intraoperative hemodynamic management was safer using the RR+WO. This observational study enlightens the RR+WO for marginal grafts in LT. However, ischemia-type biliary lesions may limit the feasibility of RR+WO. Further randomised studies should address these hypotheses.
